# Interprofessional education in cancer care – a scoping review

**DOI:** 10.1186/s12909-024-05669-8

**Published:** 2024-07-16

**Authors:** Virpi Sulosaari, Nikolina Dodlek, Andreas Brandl, Johan De Munter, Jesper Grau Eriksen, Wendy McInally, Niall O’Higgins, Kim Benstead, Celia Díez de los Ríos de la Serna

**Affiliations:** 1 Turku University of Applied Sciences, Joukahaisenkatu 3, 20520 Turku, Finland; 2 Cyprus University of Technology, Archiepiskopou Kyprianou 30, Limassol, Cyprus; 3 Department of General, Visceral and Transplantation Surgery, University of Heidelberg, Im Neuenheimer Feld 672, 69120 Heidelberg, Germany; 4 Cancer Centre University Hospital, Ghent, Belgium; 5 Dept of Experimental Clinical Oncology, Aarhus University Hospital, Aarhus, Denmark; 6 The Open University, Milton Keynes, UK; 7 School of Medicine, University College, Belfield, Dublin 4, Ireland; 8 Dept of Oncology, Gloucestershire Hospitals NHS Foundation Trust, College Rd, GL53 7AN Cheltenham, RN UK; 9 School of Nursing, University of Barcelona, Barcelona, Spain

**Keywords:** Interprofessional education, Interprofessional collaboration, Interdisciplinary education, Interspecialty training, Inter-specialty training, Oncology, Cancer care

## Abstract

**Background:**

Comprehensive cancer care requires effective collaboration by interprofessional healthcare teams. The need to develop educational initiatives to improve interprofessional collaboration is increasingly recognised. However, there is no agreement regarding the interprofessional competencies required for effective cancer care leading to much variation on the focus of research, planning and managing change. A scoping review was conducted to identify the current status of IPE in cancer care and to summarise the results of previous research in order to guide the development of interprofessional education in cancer care.

**Methods:**

The JBI Scoping Review guidelines were used to guide the process of the review. A search of the available literature was conducted in CINAHL, MEDLINE (Ovid), PubMed, PsycInfo, Scopus databases from January 2012 to March 2023 to investigate IPE for health professional clinicians working in cancer care.

**Results:**

Of the 825 initial references and 153 studies imported for screening, a total of 28 studies were included in the final review. From those studies, seven focused on the need for IPE and interprofessional competence for oncology healthcare professionals, four reviewed existing IPE programs and 17 described the development and evaluation of interprofessional education. Findings show variation and lack of concept definitions underpinning research in IPE in cancer care settings. Variation also exists in the range of research activities in IPE, most notably related to communication, teamwork and the development of interprofessional practice. The evaluation of impact of IPE is mainly focused on health care professionals’ self-evaluation and general feedback. Impact on patient care was only evaluated in one study.

**Conclusions:**

Based on the results, interprofessional education research in the field of cancer care is limited in Europe. Thus, there is a significant increase in publications in the last five years. A more systematic focus on the theoretical framework and definition of concepts would be of value. Research and programme development should be based on a shared understanding on what constitutes the interprofessional competences and IPE. Programmes to develop interprofessional practice should be developed and implemented systematically with inclusion of validated assessment methods, and evaluated and improved regularly.

**Supplementary Information:**

The online version contains supplementary material available at 10.1186/s12909-024-05669-8.

## Background

Over the last decade, there has been increased interest in developing educational initiatives to improve interprofessional collaboration and practice in the cancer care setting [1–7]. Non-communicable and life-style related diseases, including cancer, are among the biggest challenges facing EU health systems and countries, causing illness, premature death and associated social and economic costs. The number of cancer cases is expected to increase in Europe by 24% by the year 2035 [8]. As the demand in cancer care continues to increase, health systems require a workforce of educated oncology specialists and professions to provide continuity and sustainability of care. Current educational systems have yet to match all the requirements needed in cancer care [9]. Furthermore, quality cancer care requires effective collaboration by an interprofessional healthcare team [10, 11]. People with cancer benefit when the health care professionals caring for them, not only collaborate, but strive to learn from each other [12].

Interprofessional collaboration can be defined as collaborative interaction among experts with different professional backgrounds involved in care of people with cancer and who share common goals [13]. Models vary across cancer units [14] and can involve professionals from different oncology specialties (radiation, medical and surgical oncologists) and disciplines (such as pathology), professionals from nursing and social affairs and allied health professions such as physiotherapists, psychologists, nutritionists and speech therapists [15]. Professionals from varying disciplines and professions have different knowledge bases, premises and competences for cancer care and interprofessional collaboration [10, 11]. Interprofessional healthcare teams need to understand how to optimize the skills of their members, share case management and provide better health services to patients and the community. Such collaboration results in a strengthened health system and leads to improved health outcomes [12]. Furthermore, effective communication is important not only for patients but for the well-being of all healthcare professionals (HCPs) [16]. Thus, interprofessional practice requires effective leadership,administrative support [17, 18] and continuous evaluation [18].

According to The Centre for the Advancement of Interprofessional Education (CAIPE) interprofessional education (IPE) concept can be defined as occasions when two or more professionals learn with, from and about each other to improve collaboration and the quality of care [19]. The primary goal of IPE is to improve patient care by better interprofessional collaboration [6]. This concept should be reflected in the training of cancer care workforce. In oncology, the concept of multidisciplinary care is an established part of the clinical practice [20]. Training of oncology specialists and professions needs to recognise the value of interprofessional care. Interprofessional collaboration has been seen as necessary for example in precision oncology [21] and radiation oncology [22], but IPE programmes vary substantially across countries [23]. In Europe, many professional societies provide opportunities for post graduate training for medical professions. The existing training curricula of ESTRO (European Society for Radiotherapy and Oncology), ESSO (European Society of Surgical Oncology and ESMO (European Society for Medical Oncology), all recognise the importance of interdisciplinary knowledge and understanding among specialists in radiation oncology, cancer surgery and medical oncology. Yet, gaps exist in mutual understanding among the three disciplines on interprofessional practice [24]. Very few training programmes in the European curricula for oncology specialists involve formal interdisciplinary attachments or integrated interprofessional approach on cancer care. These deficits limit both the scope and the value of interdisciplinary collaboration and the drive for better care. Interdisciplinary dialogue also drives standards and improves communication [24]. 

In 2010, the World Health Organization issued a report *Framework for Action on Interprofessional Education and Collaborative Practice* stipulating that teamwork is the first among interprofessional learning domains in a clinical setting, the others being roles and responsibilities, communication, learning and critical reflection, understanding of the needs of the patient, and ethical practice [12]. Interprofessional learning (IPL) has become a more prominent feature of health professional education at both pre-qualification and post-qualification levels. While the terms interprofessional learning (IPL) and interprofessional education (IPE) may relate to differing processes, with IPL focusing more on micro learning processes and IPE being more strongly reflective of an overarching educational framework, they tend to be used interchangeably in the existing literature [25].

Recent research relates to collaborative practice skills within clinical oncology [2] and radiation oncology [6] in determination of the impact and value of interprofessional learning [26] and interprofessional communication [27]. Among the barriers to successful implementation on interprofessional education, are the variations, both in definition and in concept, underlying research on IPE. Commonly used concepts are ‘interprofessional learning’ and ‘interprofessional education’. However, recently, the European Commission has launched the concept of ‘inter-specialty training’ to combine education and training of medical, nursing and allied health professionals in the cancer care setting [28]. The concept was defined later by McInally et al. (2023): “Inter-specialty training in oncology occurs when two or more specialties within professions collaborate by learning and interacting with each other during training in order to provide high quality cancer care” [29]. However, in order to understand the context of previous studies and to inform both training programmes and future research it was considered that a scoping review was required.

The objective of this scoping review was to describe the extent and type of evidence regarding interprofessional education (IPE) in oncology. The aim was to identify how IPE has been defined, how methodology underlying the research and implementation of the IPE has been utilised, describe the state of IPE in oncology. This review is part of a European collaborative project on inter-specialty training with the intention of providing useful training programmes across Europe and beyond (INTERACT-EUROPE).

## Methodology

The current review followed the scoping review methodology. This type of evidence synthesis aims to systematically identify and map the breadth of evidence available on a particular topic, field, concept or issue. Scoping reviews can clarify key concept definitions in the literature and identify key characteristics or factors related to this concept [30]. The scoping review involved five stages: 1), Development of a scoping review protocol including research questions, and the purpose of the study; 2) Literature search on CINAHL, MEDLINE (Ovid), PubMed, PsycInfo, Scopus data bases [3], Selection of studies, 4) Data extraction, 5) qualitative analysis and presentation of results.

The questions guiding the scoping review were:

1. How has IPE been defined in previous research in cancer care?
2. What competences have been used to guide the curriculum development of IPE in cancer care?
3. What teaching, learning and assessment methods have been used in previous studies?

The PRISMA checklist extension for scoping reviews (PRISMA-ScR) was used to guide the process of the review. A detailed scoping review protocol was produced including databases and subject headings [31]. MESH terms, when applicable, were used to capture all the relevant literature. In consultation with a librarian, initial search titles and abstracts (*N* = 825) were reviewed by one researcher. All 153 items identified in this way were then downloaded into Covidence Systematic Review software (r) for further screening by three researchers. This process was followed by full text screening by the same researchers to identify articles that met the specified inclusion criteria.

### Data retrieval protocol

Databases: CINAHL, MEDLINE (Ovid), PubMed, PsycInfo, Scopus

The PCC that featured the search: P(articipants) = oncology medical professionals (medical oncologist, radiation oncologist, oncology surgeon) and nurses, C(oncept) = inter-specialty or interprofessional education or interprofessional learning, C(ontext) = Cancer care setting

### Criteria

Inclusion: Quantitative, qualitative studies and systematic reviews; papers with focus on IPE/IPL, development of interprofessional collaboration and teamwork through IPE; teaching, learning and assessment methods of IPE in the context of oncology.

Exclusion: Editorials, discussion papers, focus on other healthcare professionals outside oncology setting, non-oncological professionals, only on pre-registration students, conference abstracts and proceedings.

Limits: English language, 2012–2022 original search, updated search – to April 2023.

The main search terms: clinical oncology [MESH], medical oncology [MESH], radiation oncology [MESH], surgical oncology [MESH], oncology nursing [MESH], interprofessional training, interprofessional education, interdisciplinary education, interprofessional learning, interspecialty training, inter-specialty training.

Any conflicting screening results were discussed and decisions were made collaboratively. Full-text articles were filtered and reviewed. A data extraction sheet was pre-planned to extract the key information of the studies and reviews including authors, year, country, purpose of the study or review, study or review type and method, concept used, definition of concepts, interprofessional learning or education focus and/or competences, programme characteristics, teaching, assessment, and evaluation methods used and the main results.The data were extracted by three researchers and analysed with narrative content analysis. The process, analysis and summary of results were further discussed with the full research group including all the authors.

## Results

### Characteristics of the studies

**Fig. 1:**
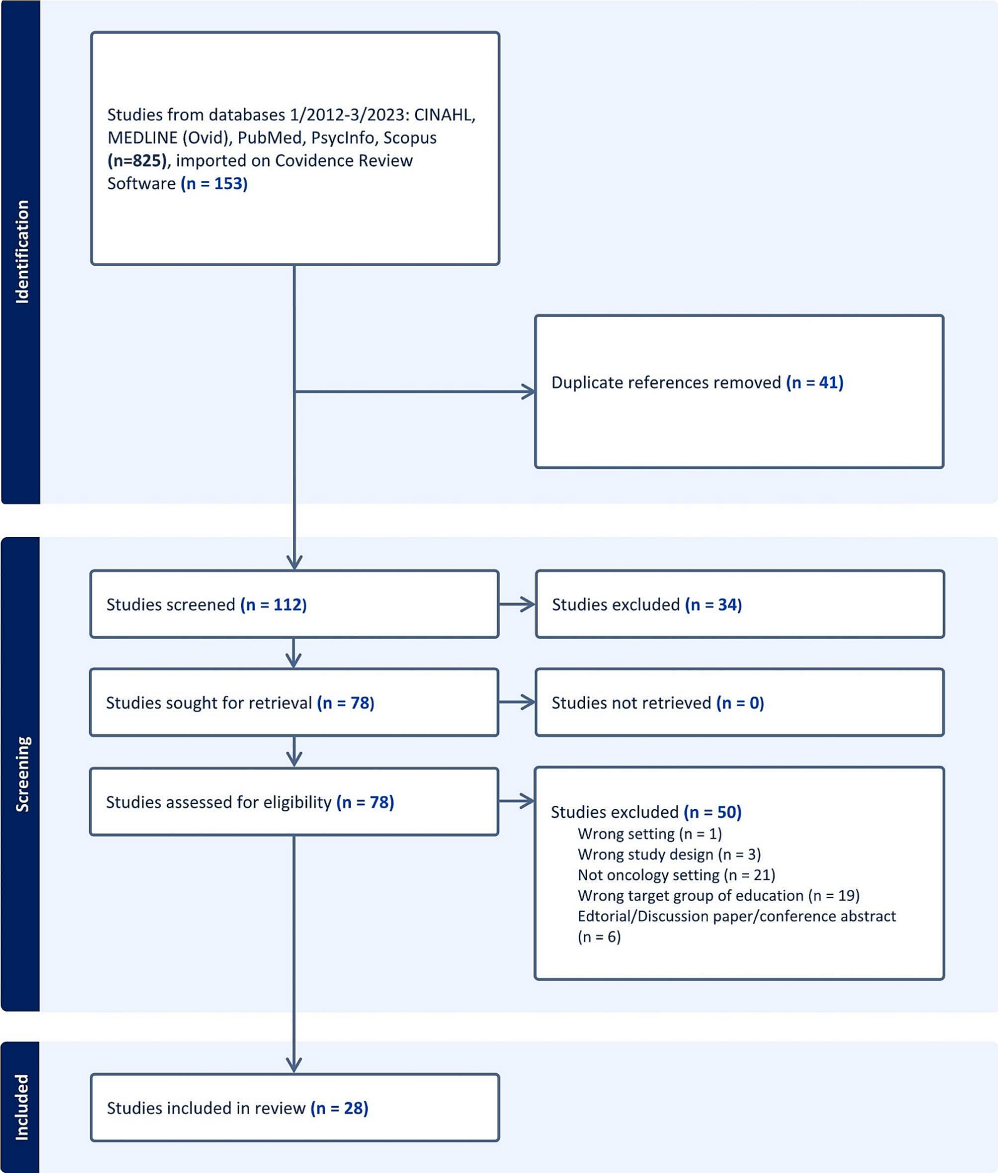
Data retrieval

A total of 28 studies were identified through database searching, of which one study was identified by reviewing reference lists (Fig. 1). There were fourteen quantitative studies, four reviews, six mixed methods studies and four qualitative studies (Table 1). The articles reported interprofessional educational programmes in United States (*n* = 15), Canada (*n* = 7), United Kingdom (*n* = 3), Denmark (*n* = 1), Germany (*n* = 1) and Switzerland (*n* = 1). Articles were published between 2012 and 2022. Nineteen of the articles were published in the last five years. From the included papers eight focused on describing the need and competences of oncology healthcare professionals [3, 4, 6, 9, 32–35], three reviewed existing IPE [1, 36, 37] or the development and evaluation (*n* = 17) of oncology interprofessional training [2, 5, 16, 22, 26, 38–49]. The target groups of the IPE included nurses, pharmacists, physicians (medical oncology, surgical oncology, radiation oncology and palliative care), radiographers, technicians and staff with healthcare backgrounds such as psychology, occupational therapy and other support workers (such as social workers, chaplaincy, or administration staff in contact with oncology patients).

**Table 1:** Summary of the reviewed papers

Author, year, country	Purpose of the study or review	Target group, setting	Type of the study	Methods
Aebersold et al., 2021, US	To describe a novel training program for oncology registered nurses and pharmacists to improve cancer drug safety	Nurses and pharmacists. Long term follow-up	Quantitative with pre-post design	**Data collection**: Questionnaires developed for the study **Data analysis**: Statistical analysis
Akthar et al., 2018, US	To quantify current interdisciplinary oncology education among oncology training programs across the USA, identify effective teaching modalities, and assess communication skills training	Oncology trainees and program directors	Quantitative; survey study	**Data collection**: A semi-structured web-based survey developed for the study **Data analysis**: Statistical analysis, qualitative content analysis (open-ended questions)
Ball et al., 2021, UK	To determine the impact and value of scenario-based learning using simulation in training of professionals working in radiation oncology	Therapeutic radiographers in postgraduate education, medical physics trainees and radiation oncology registrants	Quantitative with pre-post design	**Data collection**: Survey “Readiness for Interprofessional Learning Scale RIPLS” and a feedback questionnaire developed for the study **Data analysis**: Statistical analysis
Bunnell et al., 2013, US	To evaluate team training pilot	Physicians and nurse practitioners/physician assistants, nurses, pharmacists/pharmacy technicians and support staff	Mixed methods design; qualitative (observation, interviews) and quantitative (survey, patient incidence reports, patient satisfaction scores)	**Data collection**: Prior intervention observation and interviews of key informants to identify the problems and develop the training. Six months after implementing team training, anonymous survey developed for the study of all staff **Data analysis**: Qualitative content analysis and statistical analysis
Chollette et al., 2022, US	To examine teamwork competences or teamwork competency frameworks developed or tested in healthcare teams and understand their applicability to larger Multiteam systems MTSs	Healthcare teams in cancer care	Literature review; narrative synthesis	**Data collection**: A systematic review of relevant original articles, consensus statements and prior systematic reviews. **Data analysis**: Qualitative content analysis, narrative synthesis
Esplen et al., 2020, Canada	To describe development of a competency framework with relevance across regulated health professionals involved in cancer care	Cancer professionals (family medicine, pharmacy, social work, psychology, occupational therapy, and nursing)	Mixed methods; scoping review, qualitative study with modified Delphi technique, and a survey	**Data collection**: Seven focus group interviews, nursing-specific survey, a scoping review of national and international guidelines, standards for cancer care, and relevant competences and modified Delphi technique **Data analysis**: Qualitative thematic content analysis and statistical analysis
Gillan et al., 2015, Canada	To report on feasibility and interprofessional collaboration outcomes of a team-based simulation event	Radiation therapy, medical physics and radiation oncology trainees	Quantitative with pre-post design	**Data collection**: Surveys: Learner satisfaction and interprofessional perceptions (Readiness for Interprofessional Learning Scale (RIPLS), UWE Entry Level Interprofessional Questionnaire (UWEIQ), Trainee Test of Team Dynamics and Collaborative Behaviours Scale (CBS). **Data analysis**: Statistical analysis
Green & Markaki, 2018, US/Canada	To identify interprofessional palliative care education models applicable to pediatric oncology settings as well as methods for evaluating their impact on clinical practice	Pediatric oncology professionals	Literature review; integrative review (*n* = 13)	**Data collection**: a systematic literature search in PubMed, CINAHL and Embase **Data analysis**: Qualitative content analysis
Halm et al., 2012, US	To describe impact of an educational program	Interprofessional team members medical-surgical oncology unit, nursing, medicine, social work, chaplain, nutritionist, respiratory therapist, pharmacists, volunteers	Quantitative with pre-post design	**Data collection**: Surveys: Assessment of cultural competence using the intercultural development inventory, Frommelt Attitudes Towards Care of the Dying, knowledge of cultural beliefs/traditions, and self-perceived comfort in providing culturally sensitive end-of-life care. **Data analysis**: Statistical analysis
Harvey et al., 2020, US	To evaluate within and between group differences in self-reported confidence gains for primary care versus oncology learners across all learning objectives of the Cancer Survivorship E-Learning Series	Practiced in oncology, and primary care. Professionals: nurses, physicians, social workers and healthcare administrators	Quantitative with pre-post design	**Data collection**: Surveys developed for the study. Pre-test optional demographic survey. Post-confidence and additional questions on self-reported learning gains and intention to implement new knowledge and skills. **Data analysis**: Statistical analysis
Head et al., 2022, US	To evaluate participants’ feedback related to their experience in the Interprofessional Education Exchange (iPEX) program	Teams with representatives from at least 3 health professions, trainees completing a reflection on their learning	Qualitative study; reflective essays after the training programme	**Data collection**: Participants reflective essays **Data analysis**: Qualitative content analysis
James et al., 2016, US	To develop interprofessional team training opportunities using simulated cancer care scenarios for developing competencies in collaborative practice within the clinical oncology setting	Inpatient oncology teams (nurses and physicians)	Mixed methods; observational case study and questionnaire of participants in a cross-sectional analysis	**Data collection**: Standardised checklists for observation on teamwork skills and clinical effectiveness, post-programme evaluation surveys to determine perception of value of the training session. **Data analysis**: Descriptive, statistical analysis and content analysis (Free text responses)
Kolben et al., 2018, Germany	To assess the impact of an inhouse PC training for gynaecological nurses and physicians on competences for symptom management and palliative care	Medical and nursing staff members at a university department for gynaecology. Nurses and physicians	Quantitative with pre-post design	**Data collection**: Structured questionnaire developed for the study. Self-assessment of preparedness. Data were collected before, immediately after and 6 months after the training. **Data analysis**: Statistical analysis
Koo et al., 2014, Canada	To further investigate the attitudes of radiation oncology professionals regarding IPE and IP teaching. The secondary objective to identify the challenges faced by radiation oncologists, radiation therapists, and physicists who teach within an IP context	Clinical oncologists, physicists, radiation oncologist and radiation therapists	Quantitative; cross-sectional design with semi-structured questionnaires	**Data collection**: Questionnaire developed for the study. As part of the questionnaire some items from Readiness for Interprofessional Learning Scale, Attitudes Toward Health-Care Teams Scale, and Attitudes Toward Interdisciplinary Learning Scale. **Data analysis**: Statistical analysis, the open-ended questions thematic analysis
Laffan et al., 2015, UK	To identify the psychological training and support needs of MDT members of staff working in the oncology departments of local hospitals and the results of training	Multidisciplinary (MDT) members of oncology staff, both from within the trust and the local network	Mixed methods design; qualitative semi-structured interview and quantitative pre/post questionnaire	**Data collection**: Semi-structured interviews to identify the psychological training and support needs and questionnaire developed for the study to assess the outcome of training. **Data analysis**: Qualitative thematic analysis, statistical analysis
Lavender et al., 2014, UK	To assess the effect of radiation therapy and medical dosimetry education on knowledge and communication for radiation therapy and medical dosimetry students	Graduating students, two groups: 2007–2011, recent alumni	Quantitative; comparison design comparing previous students	**Data collection**: Students’ scores on end-of-training examinations (credentialing and exit examinations) as a measure of knowledge. Comparison of the scores with students who graduated in the 5 years prior to the initiative. **Data analysis**: Statistical analysis
McLeod et al., 2014, Canada	To report evaluation results of the first course in the IPODE project in relation to two areas: how does an interprofessional (IP), web-based, PSO (Psycho-oncology education) course influence participants’ knowledge, attitudes and beliefs about IP, person-centered PSO	Graduate students and practicing health professionals at the partner universities and agencies	Quantitative with pre-post design	**Data collection**: Semi-structured questionnaires with open-ended questions. Evaluation of the education project was guided by Kirkpatrick’s, with a focus on learner reaction and acquisition of learning. **Data analysis**: Statistical analysis. Qualitative data were analysed using directed, interpretive content analysis
Nissim et al., 2019, Canada	To evaluate a mindfulness-based group intervention, referred to as Compassion, Presence, and Resilience Training, for oncology interprofessional teams by participants subjective experience of the training and their perceptions of its benefits, risks, or challenges	Two oncology teams; participants on CPR-T training, interviews nurses, oncologists, and other oncology health professionals	Qualitative; pilot interview study	**Data collection**: Semi-structured interviews **Data analysis**: Qualitative thematic analysis
Papadakos et al., 2020, Canada	To develop and evaluate an interactive, blended simulation-based training program on supporting patients through difficult conversations	HCP trainees; nursing medicine, and radiation therapy but also included some trainees from pharmacy, dietetics, physiotherapy, respiratory therapy, and others	Quantitative design with pre-post survey	**Data collection**: Questionnaire developed for the study on participants’ competence in breaking bad news, in disclosing an incident to patients/families, and in responding to challenging motivational beliefs around having difficult conversations, self-efficacy. **Data analysis**: Statistical analysis
Pratt-Chapman, 2022, US	To assess changes to self-reported cultural competence and to examine changes to interprofessional valuation from baseline to post-intervention on Cultural Competency Training	Cancer HCP trainees; nurse, nurse practitioner, physician, navigator, administrator, social worker, other	Quantitative design with pre-post survey	**Data collection**: Cultural Competency Assessment; Lesbian, Gay, Bisexual, and Transgender Development of Clinical Skills Scale; Interprofessional Socialization and Valuing Scale. **Data analysis**: Statistical analysis
Shayne et al., 2014, US	To assess impact of a course “The Cancer Survivor Workshop”	Five teams consisting of two hematology/oncology fellows and one radiation oncology resident	Quantitative with pre-post survey	**Data collection**: Structured questionnaires (pre-post) developed for the study **Data analysis**: Statistical analysis
Shultz at al., 2021, US	To elicit qualitative reports through interviews of professionals who had completed, to characterize a need for IPE and to identify how radiation oncology professionals would structure an IPE curriculum with medical trainees as the learners	Radiation oncologists, nurses, radiation therapists, dosimetrists, medical physicists, and medical students	Qualitative; interview study	**Data collection**: Semi-structured phone interviews **Data analysis**: Thematic content analysis
Szilagyi et al., 2022, US	To improve team members’ capabilities to provide generalist spiritual care for pediatric hematology-oncology	Pediatric hematology-oncology team. 10 physicians, advanced care providers, nurse coordinators, and psychosocial team members (social workers or psychologists) providing care for AYA (age 12–25) with cancer	Quantitative with pre-post survey	**Data collection**: Questionnaires developed for the study with baseline and demographic data before and post-test measures on participants’ ability to provide generalist spiritual care, frequency of interprofessional spiritual care activities, self-efficacy and participants’ comfort with generalist spiritual care. **Data analysis**: Statistical analysis
Topperzer et all., 2019, Denmark	To identify and evaluate existing interprofessional education in pediatric	Students in postgraduate studies from more than one profession	Literature review; scoping review	**Data collection**: Systematic search on PubMed, Scopus and Education Resources Information Center. **Data analysis**: Qualitative analysis. Kirkpatrick’s modified interprofessional education outcomes model to systematise outcomes was used
Warsi et al., 2022, Canada	To evaluate the impact of workshop intervention with mentoring on knowledge regarding how to use different leadership styles, initiate changes in practice, and apply leadership skills in career development and at participant institutions	Interdisciplinary group of health professionals in cancer education with varying amounts of leadership experience in cancer education	Mixed methods; Focus group on defining the overall goals of the workshop intervention, narrative literature review to identify gaps in leadership development and determine teaching tools and program content and a pre-post survey.	**Data collection**: Review process not described in detail. A pre- and post workshop evaluation questionnaire on participants’ knowledge regarding leadership and feedback on the programme. **Data analysis**: Statistical analysis and content analysis (free text responses)
Wells-Di Gregorio et al., 2021, US	To validate core content and competencies on psychosocial oncology (PSO), particularly in underserved communities	Healthcare professionals in PMO multidisciplinary specialty	Qualitative; modified Delphi study (a single rater, absolute-agreement, two‐way mixed effects model)	**Data collection**: Phase I, a Professional Education Committee subgroup proposed domains and items, which were rated by the APOS Fellows and Board via online survey. Phase II, Fellows completed a second, revised survey. Phase III incorporated early career members. Developmental and diversity items were integrated into each domain. Phase IV, a larger group of subject matter experts were surveyed **Data analysis**: Statistical analysis
Winter et al., 2019, US	To identify reports of IPE within radiation oncology published in the literature and summarize the outcomes of those initiatives	The review targeted to find relevant literature on IPE	Literature review; systematic literature review	**Data collection**: Systematic literature search in PubMed database **Data analysis**: Qualitative analysis
Witt et al., 2020, Switzerland (multinational)	To identify and define core competences for different health professions involved in integrative oncology	Medical doctors, psychologists, nurses, naturopathic doctors, traditional Chinese medicine practitioners, yoga practitioners, patient navigators and patient advocates, public health experts, and members of the Society for Integrative Oncology	Mixed methods; systematic literature review (*n* = 21) to identify core competences and a quantitative survey	**Data collection**: Literature review, online surveys developed for the study and an onsite survey at an international integrative oncology conference. **Data analysis**: Qualitative content analysis and statistical analysis

#### Collaborative care in cancer

In oncology, medical, nursing and allied health professionals provide complex care in an interprofessional context [33, 37, 41, 45]. To provide the best treatment and care for people with cancer, healthcare professionals are required to collaborate [1, 37]. According to Head et al. (2022) interprofessional collaborative practice is an essential component of quality healthcare in oncology [42]. Effective interprofessional care was seen as necessary to provide optimal care for patients [16], improve the safety of care delivery [38] and better outcomes of patient care [16, 32, 38].

#### Terminology

Eight of the papers [3, 6, 26, 32, 34, 35, 37, 44] reported using the concept of IPE or IPL with reference to existing definition in literature. One study used the concept of “Interprofessional clinical training “ [38], one “Interdisciplinary education and training”, and one “Multidisciplinary education and training” [49], however no definition of these concepts were provided [36]. None used the concept of ‘inter-specialty or interspecialty education or training’. Nine of the included papers had some concepts described but did not include clear definitions and eleven had no mention or definition of IPE or IPL. (Appendix Table).

The concept of interprofessional education (IPE) can be seen as a means to improve health system function and delivery of care [34]. In order to achieve positive transformations in healthcare delivery, healthcare professionals (HCPs) must develop skills in interprofessional collaborative practice [42]. The principles of IPE should be embedded into every aspect of programs [36]. IPE would ideally result in greater understanding and improved communication between disciplines and professions [22, 32, 36], improved coordination [32, 36, 39], enhance team-based care management [32] and optimize more culturally affirming care [46]. Desired outcomes from IPE include also articulating one’s professional role as well as those of other professions, mutual respect, trust and willingness to collaborate [5].

#### Competency domains

In the field of oncology, increasing and building on a set of foundational knowledge, skills, and attitudes within physical, psychological, social/cultural, and spiritual domains, and collaborating with other HCPs, an early learner/novice practitioner will move towards an identity as an expert interprofessional practitioner. A competence framework on the shared set of competencies can bring professionals together, while recognizing the individuality of each profession as possessing distinct and complementary skills [9].

Of the papers describing competence framework development for interprofessional education, one focused on finding consensus on shared interprofessional competences in oncology [9], one on teamwork competences [32], one on integrative oncology [35], one on communication skills [36], one on cultural competence [46], two on paediatric oncology [1, 37], one on palliative care in oncology [42], two on psychosocial training needs in oncology [4, 33] and three papers described the specific needs of radiation oncology professionals [3, 6, 34]. Development of frameworks considered the challenges to effective coordination and the impact on patient and clinical outcomes as essential to optimal, high-quality care [32]. 

Four of the papers reported development competences for IPE. The development process was informed by guidance from an expert advisory panel with a Delphi study based on a literature review in two of the studies [9, 35]. Both Esplen and colleagues [9] and Wells-Di Gregorio and colleagues [33] started from domains proposed by an expert subgroup, Esplen and colleagues [9] incorporating also focus group interviews. In the Warsi et al. (2022) study the focus group was used to determine intervention objectives [49]. The expert panels all involved oncology professionals, and one [35] included patient and public representatives. All included shared competences divided into the domains of knowledge, skills and attitudes.

#### Participants

Target groups included in six of ten studies multidisciplinary professionals working in general oncology [16, 38, 39, 45–47], four in radiation oncology [2, 22, 26, 44], one in gynaecology-oncology [43], one in paediatrics [48] and four in different departments within the hospital or in primary care [5, 40, 41, 49]. Focus on the programmes varied. Thus, interprofessional collaboration and practice in general was included in the learning goals of the IPE in six papers [3, 6, 9, 25, 35], communication in five papers [9, 16, 26, 35, 39] and teamwork in [2, 3, 22, 26, 39, 46] representing the main areas of interest of IPE in the cancer care setting.

Five studies described existing IPE education [6, 36, 37, 42, 46], while two focused on paediatric oncology [1, 37] and one on interdisciplinary education [36]. Three of the studies used literature reviews to identify IPE [1, 37, 49] and one [36] got the information from a survey carried out by oncology physicians from different specialties.

#### Teaching methods

Teaching methods varied in methods and usefulness and included face-to-face and web-based didactic content such as lectures, workshops, educational sessions, role play and reflections. Three papers concluded that there is a lack of interdisciplinary education in oncology and also highlighted the value of IPE to professionals. (Table 2). Teaching varied in time from a one hour-long discussion group session accompanied by online modules [48] to a year-long course [44]. The mode of delivery also varied including simulated cases and scenarios (*n* = 5) [2, 16, 22, 26, 40] some specified having standardized patients [2, 16] and others were cases discussed and developed in teams [22, 26, 40] and/or by use of self-reflection [16]. Five of the studies included e-learning modules [5, 16, 38, 41, 48] alone [38, 41] or in combination with face-to-face training [5, 16, 48]; in the case of the other nine, all the training was face-to-face [2, 22, 26, 38, 40, 43–45, 47] All but one of the studies were focused on learners. One exception was based on a train-the-trainer model [39].

**Table 2:** Characteristics of interprofessional education for oncology healthcare professionals

Author, Year, Country	Aim	Design methods	Target Audience	Results	Focus (content) of the education or training / competences
Akthar et al., 2018, US	Quantify interdisciplinary oncology education among oncology training programs across the USA, identify effective teaching modalities, assess communication skills training	Quantitative, survey study	Oncology trainees and program directors: medical oncology, surgical oncology, radiation oncology, and hospice and palliative medicine	Trainees consistently reported lower rates of interdisciplinary education for each specialty. A lack of understanding regarding a collaborating specialty’s clinical care may intuitively lead to poor coordination of care, increased medical expenditures, and possibly leading to worse patient outcomes.	Not describing the training in detail, just as categories: medical oncology, surgical oncology, radiation oncology, geriatric oncology, hospice and palliative medicine, communication skills training. Geriatric oncology is the least taught discipline and communication skills as a topic.
Chollette et al., 2022, USA	To examine teamwork competences or teamwork competency frameworks developed or tested in healthcare teams and understand their applicability to larger MTSs	Systematic literature review	Practicing clinicians, students of nursing, medicine, and other allied health professionals	Half of all IPE training studies used curriculum activities that included simulation-based training plus debriefing and performance feedback to develop teamwork competences. Many studies included evaluations of teamwork competences in academic or clinical IPE training curriculums. Most of the studies focused on communication, coordination, role clarity, team-orientation, decision making and leadership.	Examined teamwork processes, teamwork competences, or team training programs implemented among healthcare providers. The most common competency domains in oncology were communication, coordination, leadership, role clarity and patient centeredness. Evaluating the effect of competency training on patient satisfaction and clinical performance.
Esplen et al., 2020, Canada	To describe development of a competency framework with relevance across regulated health professionals involved in cancer care	Mixed methods (scoping review, qualitative study with modified Delphi technique, and a survey)	Cancer professionals (family medicine, pharmacy, social work, psychology, occupational therapy, and nursing)	Competency items are structured to three levels of expertise, from early learner/novice practitioner, advancing practitioner, to expert practitioner. A set of recognized shared competences can help address challenges in team-based care and the complexities inherent in oncology.	Competency Domains of the framework: Knowledge, Skills and Attitudes. Competences: interprofessional collaboration; Recognition of the biopsychosocial and spiritual impact of cancer and the underpinnings of a person living with cancer; Foundational understanding of the cancer experiences and the cancer journey; Person-centered care, and Communication skills.
Green & Markaki, 2018, USA/Canada	To identify interprofessional palliative care education models applicable to pediatric oncology settings as well as methods for evaluating their impact on clinical practice	Integrative literature review	Pediatric oncology professionals	The data consists of 13 articles. Health professionals report receiving limited palliative care training, with little evidence of systematic evaluation of practice changes following training completion.	Essential components for successful pediatric palliative care educational programs include: [1] establishing effective modalities and teaching strategies for content delivery [2], developing an interprofessional palliative care curriculum, and [3] evaluating programs.
Head et al., 2022, USA	To evaluate participants’ feedback related to their experience in the Interprofessional Education Exchange (iPEX) program	Qualitative study	Teams with at least 3 health professionals	Improved interpersonal skills related to teamwork, increased recognition and opportunities at their home institution, improved participants’ interprofessional collaborative teaching knowledge and skills, and increased their knowledge of oncology palliative care.	Initiating IPE in home institutions with strengthening the interpersonal skills related to teamwork, knowledge of oncology palliative care, with emphasise on communication within the disciplines and teams, and the importance of learning new tools for collaborative teaching.
Koo et al. 2014, Canada	To investigate attitudes of radiation oncology professionals regarding IPE and interprofessional teaching and to identify the challenges	Quantitative, cross-sectional design with semi structured questionnaires	Clinical oncologist, physicists, radiation oncologists, and radiation therapists	A high level of agreement was received for nearly all the questions under all three domains (understanding, attitudes on IPE teaching and towards health care teams).	The understanding of Interprofessional concepts, attitudes toward Interprofessional teaching and learning environments and attitudes toward healthcare teams in needed for interprofessional teaching.
Laffan et al., 2015, UK	To identify the psychological training and support needs of MDT members of staff working in the oncology departments of local hospitals.	Mixed methods design: qualitative semi-structured interview and pre/post questionnaire	Multidisciplinary (MDT) members of oncology staff, both from within the trust and the local network	Theme for psychological training and support: Communication skills, recognizing and dealing with emotions, offering support and empathy and self-care. Participants’ overall perceived knowledge and confidence increased.	N/A
Shultz at al., 2021, US	To elicit qualitative reports through interviews to who had completed radiation oncology, to characterize a need for IPE and to identify how radiation oncology professionals would structure an IPE curriculum.	Qualitative, interview study	Radiation oncologists, nurses, radiation therapists, dosimetrists, medical physicists, and medical students	Themes identified on current IPE: Management of the radiation oncology clinic, potential impact of interprofessional training in radiation oncology, current climate of interprofessional education in radiation oncology, and creating an interprofessional training program in radiation oncology.	Interprofessional communication; patient/client/family/community-centered care; role clarification; team functioning; collaborative learning; and interprofessional conflict resolution.
Topperzer et all, 2019., Denmark	To identify and evaluate existing interprofessional education in paediatric cancer.	Scoping review	Students in postgraduate studies from more than one profession	The data consist of 9 articles. The design, strategy and content of all the studies were heterogeneous. None of the interprofessional educations systematically evaluated knowledge, skills, attitudes or the effects on patient outcomes or quality of care.	Topics: pain management and assessment, team training to prevent burnout, collaboration of healthcare professionals, training on the attitudes of healthcare professionals toward death, apheresis training and improving initiation of antibiotics for febrile patients.
Wells-Di Gregorio et al., 2021, US	To validate core content and competences on psychosocial oncology (PSO), particularly in underserved communities.	Qualitative, modified Delphi study (a single rater, absolute-agreement, two‐way mixed effects model)	Healthcare professionals in PMO, multidisciplinary specialty	Of the 394 items, 100% were rated as important to essential. No significant differences were found between disciplines on overall domain ratings.	Domains: Cancer Basics, Psychosocial Oncology, Professional Development, Ethics, Emotional/Psychological Concerns, Sexuality and Relationship Concerns, Spiritual/Religious Concerns, Healthcare Communication and Decision Making, Social/Practical Problems, Caregiver Concerns, Cognitive Concerns, Physical Symptoms and Psychosocial Assessment/Treatment.
Winter et al., 2019, US	To identify reports of IPE within radiation oncology published in the literature and summarize the outcomes of those initiatives.	Systematic literature review	The review targeted to find relevant literature on IPE targeted toward radiation oncologists, radiation therapists, nurses, medical physicists, and medical dosimetrists.	1,306 articles screened, 4 were included. Systematic review demonstrates that IPE initiatives within radiation oncology are lacking. Radiation therapists were included in IPE most frequently, followed by radiation oncologists, nurses, physicists, and dosimetrists.	N/A
Witt et al., 2020, Switzerland (multinational)	To identify and define core competences for different health professions involved in integrative oncology.	Mixed methods, literature review to identify core competences and a quantitative survey	Experts from seven different professions, patient advocates, public health experts, and members of the Society for Integrative Oncology.	The majority (80%) of the participants agreed with the suggested KSA (Knowledge, Skills and Abilities) competency categories.	Consensus procedure yielded 37 core competences relevant for all participating healthcare profession in the following categories: knowledge (*n* = 11), skills (*n* = 17), and abilities (*n* = 9).

### Evaluation

Of the 18 papers which described the evaluation of IPE programme (Table 3), 11 described also the development process [5, 22, 26, 38–41, 44, 46, 48, 49].

In the evaluation of education programmes, ten used pre- and post-programme evaluation methods [5, 16, 22, 26, 38, 40, 41, 45, 47, 48], two had mixed methods with observation and surveys [2, 39], one used qualitative evaluation with semi-structured interviews [45] and one compared the professionals participating with participants from other education activities [44]. General feedback surveys with participant satisfaction were the most common programme evaluation surveys developed for the studies.

Studies included samples of between four [44] and 1,138 participants [41]. Three of the studies included three-month follow-ups [38, 43, 48] and three studies, six-month evaluation follow-ups [43, 48, 49] indicating that gained intervention outcomes were sustained in the long term.

The following instruments were used to evaluate the impact of the IPE: (i) Readiness for Interprofessional Learning Scale [3, 22, 26], (ii) UWE Entry Level Interprofessional Questionnaire [22], (iii) Trainee Test of Team Dynamics and Collaborative Behaviours Scale (CBS) [22], (iv) Assessment of Cultural Competence using the Intercultural Development Inventory [40], (v) Frommelt Attitudes Toward Caring of the Dying [40], (vi) Attitudes Toward Health-Care Teams Scale [3], (vii) Attitudes Toward Interdisciplinary Learning Scale [3], (viii) Self-Efficacy for Interprofessional Experiential Learning Scale and End-of-Life Professional Caregiver Survey [1], (ix) Cultural Competency Assessment (CCA), Lesbian, Gay, Bisexual, and Transgender Development of Clinical Skills Scale (LGBT-DOCSS), (x) Interprofessional Socialization and Valuing Scale (ISVS) [46].

Other studies included in the review describe Delphi methods [9] and focus group interviews [4, 6, 9, 33, 39, 45] and instruments developed for the purpose of the study [5, 38, 39, 43].

Participants had positive reactions to the programmes indicating them as a promising strategy in improving cancer care [38]. They reported high levels of satisfaction [26], including improved relations within the team [22], the acquisition of new skills [41] as well as cross-cultural competence [40]. Confidence among the participants also increased [41]. Participants reported that they would highly recommend these programmes to their colleagues [2].

Participants considered that these educational events were valuable. They helped in areas such as consolidating communication, improving dialogue [5], valuing leadership [42, 44] and better understanding of spiritual needs [48]. These programmes also improved understanding of specific issues such as the effects of therapy on patients, the place of palliative care, management of pain and other symptoms and quality of life [47] and also a comprehension of the legal issues surrounding cancer [43].

There was *s*tatistically significant improvement in knowledge of teamwork principles [39] developing shared mental models, cross-monitoring situational awareness and effective conflict resolution, agreements about roles and responsibilities [22], and behaviours. Participants valued the opportunity to gain the perspective of other professions, connecting with colleagues from other disciplines practising crisis response in a simulated environment [2], and demonstrating lower levels of concern and anxiety when communicating with other professionals [44]. Some participants incorporated meditation into their daily routine by involving other family members and making it part of a “family routine” [45].

Significant improvement was also noted in increased comfort when discussing survivorship issues with patients. Significant increase in knowledge of survivorship care for five types of cancer, more confidence in ability to explain a Survivorship Care Plan (SCP), and increased comfort in discussing late effects of cancer treatment [47] were all reported. The main challenges were “breaking down the walls and being more comfortable with vulnerability” [45], and in being more open-minded after training [43]. Training increased IPE recognition of participants’ home institutions [42].

**Table 3:** Evaluation methods, description and outcomes of the IPE

Author Year Country	Methods	Description of the IPE	Outcomes
Aebersold et al. 2021, US	*Design*: Pre-post questionnaires *Sample size*: 521 *Data Collection Methods*: Questionnaires developed for the study: (a) pre- and post- knowledge test, (b) program satisfaction survey, (c) Intention to changes	*Learning Goal*: Improve the safety of cancer drug delivery and interprofessional collaboration. *Teaching Methods*: Pre-learning modules and workshop delivered by interprofessional faculty. *Topics / Competences*: modules on six topics: hazardous drug handling, drug extravasation, hypersensitivity reaction management, sepsis recognition, immune checkpoint inhibitor toxicities and oral oncolytic adherence. *Duration*: 7-hour workshop.	Confidence increased between pre- and post workshop (range of increase 0.6–0.8). Knowledge increased significantly between pre- and postworkshop (average improvement of 3.2 points, *p* = .01). Overall program satisfaction was high (mean 5.0, standard deviation SD 0.2 on a five-point scale).
Ball et al., 2021, UK	*Design*: Pre-Post Questionnaires. *Sample size*: 26 *Data Collection Methods*: Survey RIPLS and a feedback questionnaire developed for the study	*Learning Goal*: Enhance participants’ teamwork and collaboration skills. *Teaching Methods*: 4 radiotherapy scenarios within a simulation. *Topics / Competenies*: Communication skills, consenting patients lacking capacity to consent, creating an immobilisation shell, interdisciplinary communication, patient care, team working, radiotherapy “skin apposition” setup, group decision making, error check, clinical reasoning, applying correct process for reporting, calculating adjustment to dose and fractionation to compensate, *Duration*: One day	Participants reported high levels of enjoyment related to collaborative working, communication and observing other professionals deploying their technical skills and specialist knowledge. Readiness for interprofessional learning increased in Pre-post measurement (range of increase 74,96 − 78,58, *p* = .0025).
Bunnell et al., 2013, USA	*Design*: Mixed methods. *Sample size*: 106 *Data Collection Methods:* qualitative (observation, interviews) and quantitative (survey, patient incidence reports, patient satisfaction scores).	*Learning Goal*: to reduce the risk of error in the processes of ordering, dispensing and administering chemotherapy and to improve the practice environment and team satisfaction through the more effective team communication and collaboration. *Teaching Methods*: Team training. Train-the-trainer model. *Topics / Competences*: Team training principles and supporting data (the role of leadership, communication techniques, developing shared mental models and situational awareness, and error reduction). *Duration*: 16 h.	Statistically significant improvement in knowledge of teamwork principles. The incidence of missing orders for unlinked visits decreased from 30–2%. Patient satisfaction scores regarding coordination of care improved from 93 to 97. Providers, infusion nurses and support staff reported improvement in efficiency, quality and safety, and more respectful behaviour and improved relationships among team members.
Gillan et al., 2015, Canada	*Design*: Pre-Post Questionnaires. *Sample size*: 21 *Data Collection Methods*: Learner satisfaction and interprofessional perceptions RIPLS, UWEIQ, Trainee Test of Team Dynamics and collaboration Behaviours Scale CBS.	*Learning Goal*: Clinical situations requiring significant and often rapid medical decision. *Teaching Methods*: Team-based simulation (five cases). *Topics / Competences*: The case scenarios reflected unique situations that would ideally require engagement of all three professions. *Duration*: three 105-minute timeslots	All scores improved. Perceptions of team functioning and value of team interaction in ‘establishing or improving the care plan’ were high for all cases. RIPLS and UWEIQ scores reflected positive perceptions both pre- and post-event. CBS scores were 70·4, 71·9 and 69·5, for the three cases, scores increasing between the first and second case for 13/21 (61·9%) participants. RIPLS and UWEIQ scores reflected positive perceptions both pre- and post-event, averaging 83·5 and 85·2 (RIPLS) and 60·6 and 55·7 (UWEIQ).
Halm et al., 2012, US	*Design*: Pre-Post Questionnaires. *Sample size*: 30 *Data Collection Methods*: Post programme *s*urvey, Intercultural Development Inventory, Attitudes Towards Care of the Dying, knowledge of cultural beliefs/traditions, and self-perceived comfort.	*Learning Goal*: To improve knowledge and attitudes of interprofessional team members toward culturally mediated end-of-life beliefs and practices and their comfort in providing such care in the immediate period following the intervention. *Teaching Methods*: 2-stage educational program *Topics / Competences*: From the framework Swanson’s theory of caring: Knowing the patient, Being with, or emotionally present, Doing for, Enabling and maintaining belief. *Duration: N/A*	The t tests showed no significant differences between pre-post attitude and knowledge scores (*P* > .05). Despite these findings, staff’s perceived level of understanding of end-of-life care beliefs, preferences, and practices of the Latino, Russian, and Micronesian cultures, as well as comfort and effectiveness in providing culturally sensitive end-of-life care, were higher after the in-service and critical reflection sessions.
Harvey et al. 2020, US	*Design*: Pre-Post questionnaires. *Sample size*: *N* = 985 *Data Collection Methods*: Surveys Pre- and post- confidence + self-reported learning gains and intention to implement.	*Learning Goal*: Provide education and training to primary care and oncology professionals on cancer survivorship care. *Teaching Methods*: E-learning modules. *Topics / Competences*: Managing late and long-term effects, addressing health behaviours, addressing social and emotional needs, coordinating interprofessional care, and providing evidence-based clinical care management. *Duration: N/A*	Participants gained new skills/strategies they could apply to practice. Most of the participants (85.4% oncology, 84.3% primary care) enhanced their knowledge and that they planned to implement skills/strategies into practice (77.5% oncology, 79.1% primary care).
Head et al., 2022., US	*Design*: Pre-post Questionnaires *Sample size: N = 78* *Data Collection Methods*: Questionnaires developed for the study: pre- and post and programme evaluation	*Learning Goals*: Initiating IPE in home institutions *Teaching methods*: Face-to-face workshop, video calls, role play, small group exercises, didactic teaching. *Topics/Competences*: Interpersonal skills related to teamwork, knowledge of oncology palliative care. *Duration*: 4 months	*Reaction*: iPEX participation improved their working in a team rather than as an individual. iPEX participation improved interpersonal skills related to teamwork, increased their recognition and opportunities at their home institution and increased knowledge.
James et al. 2016 US	*Design*: Mixed methods. *Sample size*: 23 *Data Collection Methods*: Standardized checklists on teamwork skills and clinical effectiveness and survey: Post-programme evaluation surveys	*Learning Goal*: Develop interprofessional team training opportunities and develop competences in collaborative practice. *Teaching Methods: S*imulated cancer care scenarios. *Topics / Competences*: Selected cases included cancer-related anaemia, extravasation of chemotherapy, and acute delirium in the inpatient oncology setting. *Duration: N/A*	Participants valued the opportunity to gain the perspective of other professions, connect with colleagues from other disciplines, and practice crisis response in a simulated environment. Practice skills in oncology-specific tasks were rated with high values.
Kolben et al. 2018, Germany	*Design: Quantitative* *Sample size*: 27 *Data Collection Methods*: Pre and post questionnaire, open questions on working experience, personal motivation, suggestion for improvement and self-assessment	*Learning Goal*: Gain knowledge on aspects of palliative care, improved the attitude towards the interaction with other disciplines and towards palliative care. *Teaching Methods*: Psycho-oncological aspects of communication with cancer patients and their relatives, symptom control, wound management and interdisciplinary communication. *Duration*: 17 h over three months	Improved physicians’ and nurses’ understanding of palliative care and their core competences in pain and symptom management, communication and understanding of legal aspect. A more positive attitude towards palliative care in the majority of participants right after the workshop: (62%), 6 months after: (71%).
Lavender et al. 2014; UK	*Design*: Quantitative *Sample size*: 44 *Data Collection Methods*: Scores end-of-training examinations. Questions to assess attitudes and willingness to communicate	*Learning Goals*: All members of the team should be able, and expected, to work together, share ideas and knowledge. *Teaching methods*: educational conference, monthly morbidity and mortality conferences, monthly quality assurance conferences, daily peer-review sessions, daily departmental huddle, chart rounds. *Topics/Competences*: Team building through interprofessional education and interprofessional collaboration. *Duration*: One year, three times per week	Students who attended the IPE sessions were more comfortable speaking with attending physicians, residents, physicists, and faculty. Mean score on the therapy credentialing examination from 2007 to 2011 was 82.7% vs. 87.5%, in 2012.
McLeod et al.2014. Canada	*Design: Q*uantitative pre-post design *Sample size: 210* *Data Collection Methods*: Pre and post questionnaire that explored how an interprofessional, web-based, PSO course influenced participants’ knowledge, attitudes and beliefs about IP.	*Learning Goals*: Mutual respect, trust and willingness to collaborate with other professions. *Teaching methods*: In-depth cases and videos, reading, discussion board, online content. *Topics/Competences: S*pecialty education, attitudes and beliefs about IP, person-centered PSO care. *Duration: N/A*	Course participants highlighted a variety of ways in which the course expanded their vision about what constitutes an IP team and increased their confidence in interacting with healthcare professionals from professions other than their own. Statistically significant difference was identified on posttest on culture difference among professions (p .<05), confidence on interacting with others (p .<05) and understanding of other professionals roles (*p* < .01).
Nissim et al., 2019 Canada	*Design*: Qualitative pilot study *Sample size: 10* *Data Collection Methods: S*emi-structured interviews exploring participants’ experience of the CPR-T. With questions about perceived benefits or challenges during the CPR-T and afterward.	*Learning Goals*: Cultivate compassion, responsiveness, and self-care; strengthen presence, focused attention, and calm and non-judgmental acceptance; and build resilience, stamina, balance, and the ability to face stressful circumstances. *Teaching methods*: Two pilot groups in face-to-face sessions. *Topics/Competences*: Self-care, self-compassion, organizational acknowledgment and recognition of stress *Duration*: 8 weeks- 1.5 h per week.	Some participants incorporated meditation into their daily routine by involving other family members and making it part of a “family routine.”
Papadakos et al., 2020 Canada	*Design*: Mixed methods pre-post questionnaires *Sample size: 64* *Data Collection Methods*: Online survey.	*Learning Goals*: Improvement of participants’ confidence in breaking bad news and communication. *Teaching methods*: Simulation, online theoretical learning and reflective practice in addition to in-person simulation with standardised patient actors, short videos followed by discussion. *Topics/Competences*: Communication skills, speaking in plain language, tips for breaking bad news, incident disclosure, resilience and coping. *Duration*: N/A	Participants rated their confidence in mastering the techniques they learned with an average score of 71 ± 16 points, with 43% of participants rating their confidence at 75 or higher. *Behaviour: S*elf-perceived competence ratings significantly increased from an average of 42 ± 22 points before to an average of 67 ± 15 points after. 93% of participants experienced an increase in their competence ratings.
Pratt-Chapman, 2022., US	*Design*: Mixed methods survey, pre-post design *Sample size: 47* *Data Collection Methods*: Pre and post survey. Cultural Competency Assessment (CCA), scale LGBT-DOCSS and Interprofessional Socialization and Valuing Scale (ISVS)	*Learning goals*: Implementation of quality improvements to advance equitable, accessible, and patient-centered cancer care. *Teaching methods*: Virtual technical assistance sessions and in person workshop, no specific methods defined. *Topics/competences*: Improved patient-provider communication, cultural sensitivity, shared decision-making, and attention to health literacy. *Duration*: 5-hour online course followed by 2.5-day in-person workshop.	Higher cultural behaviours and attitudinal awareness about LGBT health. Significant improvement to learners’ self-reported cultural competence to care for patients of diverse racial, ethnic, sexual, gender, geographic, and other intersectional lived experiences ( Cultural Competency Behaviors [*p* = .055], a subscale of the CCA, and Attitudinal Awareness toward sexual and gender minorities [*p* = .046], a subscale of the LGBT-DOCSS, using [*p* < .10)].
Shayne et al., 2014 US	*Design*: Pre-post survey, quantitative data (Likert scale). *Sample size: 15* *Data Collection Methods*: Pre-post questionnaire with Likert scale	*Learning Goals*: To establish a collaborative effort between the disciplines of hematology/oncology and radiation oncology. *Teaching Methods*: Case reports, discussion on peer-rewieved articles. *Topics/Competences*: Learning about survivorship from patient, primary care physician, and oncologist perspectives using a curriculum based on survivorship literature; designing treatment summaries and survivorship care plans. *Duration: N/A*	Improvement was noted in comfort discussing survivorship issues with patients (*p* = .001), reported knowledge of survivorship care for five types of cancer (*p* = .002), confidence in ability to explain a Survivorship Care Plan (*p* = .001), and comfort discussing late effects of cancer treatment (*p* = .001).
Szilagyi et al., 2022., US	*Design*: Pre-post survey, mixed methods *Sample size: 21* *Data Collection Methods*: post-test measures at 1, 3, and 6 months after the intervention.	*Learning Goals*: To improve team members’ capabilities to provide generalist spiritual care for pediatric hematology oncology patients. *Teaching methods*: Six in-person, hour-long discussion group sessions accompanied the six online ISPEC modules *Topics/Competences*: Spiritual care, frequency of interprofessional spiritual care activities, self efficiacy, participants’ comfort with generalist spiritual care. *Duration*: Six in-person, hour-long in person + online modules.	Ability to provide generalist spiritual care increased by 36%. Outcomes were sustained in the long term.
Warsi et al.,2022., US	*Design*: Mixed methods, pre-post survey *Sample size*: 15 *Data collection methods*: First, a focus group, second literature review, third, pre-post survey developed for the study on evaluation of the course.	*Learning goals*: Developing leadership skills; mentorship and scholarship. *Teaching methods*: Lectures/seminars for foundational content, case-based activities, networking sessions, individual learning projects, presentations by participants, and mentorship, breakout sessions in small groups. *Topics/competences*: Leadership, management, building successful cancer education *Duration*: In person one-day workshop.	Participants reported an increase in knowledge regarding how to use different leadership styles, initiate changes in practice.

Legend: (Readiness for Interprofessional Learning Scale (RIPLS) Level Interprofessional Questionnaire (UWEIQ), Collaborative Behaviours Scale (CBS)

## Discussion

To the best of our knowledge, this work is the first scoping review summarising the existing research on interprofessional education and learning in the cancer care setting. We identified 28 articles published between January 2012 and March 2023 with a significant increase in publication in the last five years. The results indicate growing interest of interprofessional education in this setting. Interestingly, most of the studies and reviews identified were from US and Canada, showing the need for further research and collaboration in Europe.

This highlights and strengthens the need for collaborative initiatives and projects such as INTERACT-EUROPE, launched in 2022. The project is based on the EU Beating Cancer Plan 2021 in which the concept of “inter-specialty training” was launched to combine education and training of medical, nursing and allied health professionals in the cancer care setting [27]. This extends the use of specialty to also include different professionals, not only specialties among one profession [29]. The EU Beating Cancer Plan is a key strategy document for cancer care development across Europe, including the training of cancer care workforce. The concept will be used in the European training programmes. Therefore, it was important to understand its similarities and differences with most used concepts, especially, interprofessional education (IPE). One important aim of the review was to identify the concepts used in research and IPE development in the field. We found that *interprofessional education* [3, 4, 6, 9, 16, 22, 26, 33, 36, 38–43, 45] is the most common term used. However, it seems that, in the oncology setting, there is need for improving the theoretical underpinnings of education research on interprofessional education. Inter-specialty training and interprofessional education are very similar. Thus, inter-specialty as a concept could refer to education including only specialities of one profession. To enhance the interprofessional practice it is, however, important to be inclusive for all professionals of the multidisciplinary teams. From 11 studies and reviews using the IPE or IPL, only eight studies defined or described the concept. In the McInally et al., (2023) study, medical doctors and nurses from the oncology field, expressed lack of understanding of the inter-specialty training as a concept [29]. IPE may have been more familiar to participants; however, the results of the study and our review indicate that conceptual clarity is needed in future studies and development of inter-specialty training.

The illustrates the multidisciplinary context of cancer care settings and the need for professionals to work together for people with cancer to achieve optimal care outcomes [10, 11, 50]. Interprofessional education, bringing the professionals together, can improve interprofessional practice and collaboration. According to our results, participants evaluate IPE positively and improving the collaboration. To support the development of interprofessional practice in oncology, as described in the WHO Interprofessional Collaborative Practice framework, describing and defining the common competences for inter-specialty training programme is important [12].

The shared set of competences in oncology practice included interprofessional collaboration, recognition and understanding or needs and experiences of a person affected by cancer, person-centred care and service approach, communication skills, use of technology in care delivery and understanding of one’s own limits and ability for self-care. James et al., (2016) also highlighted the values and ethics, roles and responsibilities [2] and Koo et al., (2014) attitudes towards interprofessional collaboration, in addition to communication competences and teamwork capabilities [3]. The above-mentioned are also key elements of interprofessional education. The aim is not for learners to master all the disciplines and professional expertise, but more about understanding the expertise of different professions and what they need to know about the specialty or the profession to work efficiently together [2]. Thus, our review provides information also on the common foci of the programmes in addition to more general interprofessional practice and team work focus; care and symptom management [2, 22], paediatric oncology (1, 36, 47 ), safety [38, 39], psycho-social oncology and psycho-oncology [4, 5, 33, 43], palliative and end-of-life care [1, 40, 43], spiritual care [48], integrative oncology [35], survivorship [41, 47] and well-being at work of healthcare professionals [45]. Although, our review focused on oncology, findings on theoretical underpinnings of the studies and programmes, teaching methods and evaluation of IPE could be used on other specialties also.

A variety of methods was used on the IPE. The content and focus and learning goals of IPE, and what constitutes interprofessional education varied between the studies and reviews. This has been highlighted also as common to IPE by the Committee on Measuring the Impact of Interprofessional Education on Collaborative Practice and Patient Outcomes (2015) [51]. Programme evaluation was mostly limited on participant satisfaction and feedback on delivery of the education. The evaluation methods mostly used in evaluation of the impact of education interventions were knowledge tests, behaviour change, confidence, comfort, intention to change practice and self-assessment of preparedness, Previously validated instruments were used in five studies [3, 22, 26, 40, 46], but it was common to use survey instruments developed for the study. Impact on patient care was not often measured. In one study patient incidence reports were included [14]. This result demonstrates a need for more systematic use of previously developed evaluation and assessment methods when appropriate, but also extent the evaluation of impact on care.

### Limitations

A limitation of this study is that only publications in English were included. Neglecting the potential data of studies from non-English speaking countries can have an impact on the results. Furthermore, we used broad subject headings with Boolean operators in search of literature, but it is possible that we did not find all the papers published. To decrease this risk, a professional librarian was consulted in the literature search process. We did not include studies focusing only on one specific cancer type, and this also needs to be seen as a limitation. As is common in scoping reviews, the quality of publications was not assessed, and we included papers with a variety of methods in the review. This needs to be recognised in interpreting the results. Thus, this review approach produced more rich data for describing the current state of IPE in cancer care.

## Conclusions

Based on the review, research on interprofessional education in the field of cancer care is limited. The need for interprofessional education is well recognised, yet provision and research in this field needs to be increased to enhance quality, person-centred care for people affected by cancer and efficient delivery of cancer care. In the future research would benefit from a more systematic approach to underpinning the theoretical framework on IPE. The evaluation of impact of IPE is currently mainly focused on HCPs perspective. Further research is needed to evaluate the impact on patient care. It is also evident that research and IPE programme development is very limited in the European context and therefore research is needed to strengthen the IPE development in Europe.

### Electronic supplementary material

Below is the link to the electronic supplementary material.

Supplementary Material 1

## Data Availability

All data generated or analysed during this study are included in this published article. Clinical Trial Number: N/A.

## Abbreviations

IPE: Interprofessional Education
IPL: Interprofessional Learning
HCP: Healthcare Professional
