# Supplementary material for: Interprofessional education in cancer care – a scoping review
Source: BMC Med Educ. 2024 Jul 16;24:767. doi: 10.1186/s12909-024-05669-8 (PMC11253347; doi:10.1186/s12909-024-05669-8)
Supplement: Supplementary file 1 — Supplementary Material 1 [file 12909_2024_5669_MOESM1_ESM.docx]

Supplementary material table: IPE definitions used in the papers.

| **Author, year, country** | **Concept and/or definition of Interprofessional education or learning (IPE/IPL)** | **Description of the interprofessional education or learning** |
| --- | --- | --- |
| **Aebersold et al., 2021, US** | Interprofessional clinical training, no definition available. | It is essential to embrace interprofessional clinical training to improve the safety of care delivery and outcomes of patient care. |
| **Akthar et al., 2018, US** | Interdisciplinary education and training, no definition available. | Enhanced interdisciplinary education would ideally result in greater understanding and improved communication between disciplines, more timely and appropriate referrals, greater consensus at multidisciplinary tumour boards, and improved coordination. |
| **Ball et al., 2021, UK** | Interprofessional learning to enable students to learn about other key roles within their sphere of healthcare and the responsibilities of all concerned, whilst still maintaining their own distinctiveness and having an appreciation for the inter-dependability of staff needed for complete healthcare provision; all with the ultimate aim of improving patient care. | Key aspect of interprofessional learning is group work that draws on individual professional knowledge to develop enhanced understanding of others' roles and responsibilities. |
| **Bunnell et al., 2013, USA** | No definition available. | Oncology care is delivered largely in ambulatory settings by interdisciplinary teams. Effective interdisciplinary and interprofessional communication and coordination for the successful care of the patient are a necessity in outpatient oncology care. |
| **Chollette et al., 2022, USA** | Interprofessional education: IPE is defined as educators and  learners of two or more health professions and their foundational  disciplines who jointly create and foster a collaborative learning environment [Buring et al. 2009]. | IPE provides an opportunity for teamwork skill development to enhance team-based care management. Training in team processes and competencies such as collaboration, communication and coordination is often a key component of IPE teamwork training. Teamwork competency frameworks or IPE curriculums specifically address teamwork among larger, distributed teams or examined competencies are necessary to overcome the care coordination challenges in cancer care. Development of frameworks and IPE curricula considers challenges to effective coordination and the impact on patient and clinical outcomes as essential to optimal, high-quality care. |
| **Esplen et al., 2020, Canada** | No definition available. | The created framework on the shared set of competencies brings professionals together, while recognizing the individuality of each profession as possessing distinct and complementary skills. By increasing and building on a set of foundational knowledge, skills, and attitudes within physical, psychological, social/cultural, and spiritual domains, as well as by collaborating with other health professionals, an early learner/novice practitioner will move towards an identity as an expert interprofessional practitioner in the field of oncology. |
| **Gillan et al., 2015, Canada** | Interprofessional education, no definition available. | Interprofessional collaboration is increasingly being recognized in medical trainee competency. In teaching interprofessional collaboration, IPE exercises were found to lead to common clinical terminology, better consideration of professional perspectives and knowledge, and enhanced communication. |
| **Green & Markaki, 2018, USA/Canada** | Interprofessional education, no definition available. | Interprofessional approach requires highly-skilled team members working together. |
| **Halm et al., 2012, US** | No definition available. | N/A |
| **Harvey et al., 2020, US** | No definition available. | There is a need for multidisciplinary healthcare providers competent in addressing complex healthcare needs. |
| **Head et al., 2022., USA** | No definition available. | Interprofessional collaborative practice is an essential component of quality healthcare. To become an effective team member, IPE is necessary. The principles of IPE should be embedded into every aspect of the program. |
| **James et al., 2016, US** | Interprofessional education, no definition available. | In order to achieve positive transformations in healthcare delivery, healthcare professionals must develop skills in interprofessional collaborative practice. |
| **Kolben et al., 2018, Germany** | Interdisciplinary training, no definition available. | N/A |
| **Koo et al. 2014, Canada** | Interprofessional education: IPE occurs when students from two or more professions learn about, from and with each other to enable effective collaboration and improve health outcomes. IPE enhances learners’ understanding of other professions’ roles and responsibilities, while fostering mutual respect and understanding between members of the healthcare team [Curran et al. 2007]. | N/A |
| **Laffan et al., 2015, UK** | No definition available | N/A |
| **Lavender et al. 2014, UK** | Interprofessional education and interprofessional learning: IPE domains are teamwork, roles and responsibilities, communication, learning and critical reflection, relationship with and recognizing the needs of the patient, and ethical practice [WHO Framework for Action on Interprofessional Education and Collaborative Practice, 2010] | All members of the team should be able to work together, share ideas and knowledge, thus fostering team-based learning. |
| **McLeod et al., 2014, Canada** | Interprofessional education, no definition available | Desired outcomes from IPE include articulating one’s professional role as well as those of other professions, mutual respect, trust and willingness to collaborate. |
| **Nissim et al., 2019 Canada** | No definition available. | In oncology, medical, nursing and allied health professionals provide complex care in an interprofessional context. |
| **Papadakos et al., 2020, Canada** | No definition available | Effective interprofessional care is necessary to provide optimal care for patients. |
| **Pratt-Chapman, 2022, USA** | No definition available | The effectiveness of the TEAM training among an interprofessional group is important, because building competence among diverse professionals within an organization provides trainees with allies that may optimize more culturally affirming care at multiple level. |
| **Shayne et al., 2014, US** | No definition available | N/A |
| **Shultz at al., 2021, US** | Interprofessional education: IPE typically involves students from 2 or more medical professions learning about, from and with one another. Interprofessional collaboration is the ultimate goal of IPE. | IPE is gaining recognition as a means of improving healthcare delivery and patient outcomes. IPE was based on the CIHC framework and Barr et al’s IPE-specific interpretation of Kirkpatrick’s hierarchy of learner outcomes. |
| **Szilagyi et al., 2022, US** | Interprofessional education, no definition available. | N/A |
| **Topperzer et al., 2019., Denmark** | Interprofessional education: IPE can be defined as occasions when two or more professionals learn with, from and about each other to improve collaboration and the quality of care [Freeth et al. 2005; original definition from The Centre for the Advancement of Interprofessional Education, CAIPE]. | Complex treatment, care and rehabilitation require collaboration between healthcare professionals. To provide the best treatment and care, healthcare professionals are required to collaborate. |
| **Warsi et al., 2022, USA** | No definition available. | Members of an interdisciplinary healthcare team have unique responsibilities related to their role. Leadership development in cancer education for oncology professionals will improve interprofessional collaboration, facilitate change for the benefit of patients and foster productive workplaces. Implementing changes in practice as interdisciplinarity in leadership aims to optimize patient care. |
| **Wells‐Di Gregorio et al., 2021, US** | Multidisciplinary education and training, no definition available. | Multidisciplinary specialty including physicians, nurses, psychologists, social workers, and other experts. |
| **Winter et al., 2019, US** | Interprofessional Education: IPE occurs when learners from two or more professions learn about, from and with each other to enable effective collaboration and improve health outcome (WHO, 2010). | The concept of interprofessional education (IPE) can be seen as a  means to improve health system function and delivery of  care. |
| **Witt et al., 2020, Switzerland** | Any educational initiative designed to improve understanding or teamwork efficiency between the learners of interest, no clear definition available. | N/A |
